# Exploring the diagnostic accuracy of an HIV self-test optimized by a digital app-based solution: Results from a secondary data analysis of a field trial in South Africa

**DOI:** 10.1371/journal.pdig.0000791

**Published:** 2025-04-08

**Authors:** Ashlyn Beecroft, Aliasgar Esmail, Olivia Vaikla, Thomas Duchaine, Nora Engel, Chen Liang, Qihuang Zhang, Keertan Dheda, Nitika Pant Pai

**Affiliations:** 1 Division of Experimental Medicine, Department of Medicine. McGill University, Montreal, Quebec, Canada; 2 University of Cape Town, Cape Town, South Africa; 3 Research Institute of the McGill University Health Centre, Montreal, Quebec, Canada; 4 Department of Medicine, McGill University, Montreal, Quebec, Canada; 5 Department of Health, Ethics & Society, Care and Public Health Research Institute, Maastricht University, Maastricht, The Netherlands; Makerere University CHS: Makerere University College of Health Sciences, UGANDA

## Abstract

**Background:**

To reach UNAIDS 95-95-95 targets, digital HIV self-testing (HIVST) strategy aided by applications, platforms, and readers can engage young people and adults living with undetected HIV infection. Evidence on its acceptability, feasibility, impact exists, yet accuracy data are limited.

**Methods:**

A secondary data analysis of a quasi-RCT of digital HIVST in South Africa was performed. We hypothesized app-guided digital interpretation of oral self-test enhanced test accuracy. We compared accuracy between digital HIVST supervised vs. unsupervised (with/without healthcare worker). Self-test results were interpreted and uploaded by participants, compared using computer vision technology, against lab reference standard by trained healthcare professionals.

**Results:**

1513 digital HIVST participants reported pooled Sensitivity (Sn) = 95.52% (95% CI, 94.48%-96.56%); Specificity (Sp): 99.93% (95% CI, 99.79%-100.06%); Positive predictive value (PPV): 99.22% (95% CI, 98.78%-99.67%); Negative Predictive Value (NPV): 99.57% (95% CI, 99.24%-99.90%). 565 participants on supervised digital HIVST, reported a pooled Sn: 93.65% (95% CI, 91.64-95.66); Sp: 100.00% (95% CI, 100.00-100.00); PPV: 100.00% (95% CI, 100.00-100.00); NPV: 99.21% (95% CI, 98.48-99.94). 968 unsupervised digital HIVST participants, reported a pooled Sn: 97.18% (95% CI, 96.13-98.24); Sp: 99.89% (95% CI, 99.67-100.10); PPV: 98.57% (95% CI, 97.82-99.33); NPV: 99.77% (95% CI, 99.47-100.08). Non-digital HIVST vs. study digital HIVST data at 5% significance level - Sn: chi = 0.6495, p-value = 0.4203, Sp: chi = 0.3831, p-value = 0.5259. Supervised vs. unsupervised HIVST at 5% significance level - Sn: chi = 0.973, p-value = 0.3237, Sp: chi = 0.527, p-value = 0.4449.

**Conclusions:**

Digital HIVST improved interpretation of test results, increased accuracy and predictive value estimations (upper limit 98%-100%), removing subjectivity. Unsupervised digital HIVST users performed better than supervised. Digital HIVST results can potentially signal a rapid triage to therapy or prevention pathways, while awaiting lab confirmation. Findings have implications for scale up of digital HIVST initiatives in global settings.

## Author summary

The reason I conducted this research is to bring light to the improvement in accuracy that can be achieved with digital supports in the self-testing process. HIV is a prominent stigmatized illness that can be difficult for patients to receive care for while avoiding prejudice. Digital HIVST is a way that can reach those who are afraid to seek care which will increase HIV status awareness while also increasing regularity of testing which will hopefully diminish stigma. With the COVID-19 pandemic, we all saw how impactful self-testing can be, and to apply this important work in an area of health that needs it due to preconceived notions and shame could improve the lives of millions worldwide.

## Introduction

The human immunodeficiency virus (HIV) pandemic has impacted the lives of 38.4 million individuals worldwide [1]. Despite advances in testing and diagnosis, one out of every five individuals are unaware of their positive HIV serostatus and can unknowingly transmit the virus [2]. To end the spread of HIV infection by 2030, the United Nations Programme on HIV/AIDS (UNAIDS) has set a 95-95-95 target to be reached by the year 2025 [3]. These targets state that by 2025, 95% of people living with HIV (PLWH) will know their status, 95% of those who know their status will initiate treatment, and 95% of those who initiate treatment will have suppressed viral loads [3]. HIV self-testing (HIVST) is a last mile solution with sufficient evidence from the African subcontinent, to increase the general population’s knowledge of HIV serostatus and has been proposed as a trusted method from health policy makers. Individuals can self-test from the comfort of their own home, thereby increasing accessibility of testing. Prior systematic reviews reported high acceptability, feasibility, and uptake of HIVST among key populations including men who have sex with men (MSM), sex workers (SW), people who inject drugs (PWID), transgender people, and people in prisons or closed settings [4–6]. In a systematic review, Pant Pai and colleagues reported high acceptability and preference rates among participants ranging from 74%-96% and 61%-91%, respectively [4]. Figueroa et al. analyzed acceptability, defined as “the willingness to take a test in the future or as an increased frequency of testing with a HIV home-test”, and reported rates above 67% [5]. A meta-analysis conducted by Witzel et al. examined the effects of HIVST compared to standard HIV testing methods for key populations [6]. They found that HIVST increased test uptake by an average of 1.45 times (RR=1.45, 95% CI 1.20-1.75) [6]. As well, HIVST increased the mean number of HIV self-tests among MSM and transgender people by 2.56% (95% CI, 1.24-3.88) [6].

Recently, digital HIVST has risen in popularity [7]. The authors reported a high acceptability (91%; range 87-95%) among?? of social media and mobile app-based HIVST methods worldwide [7]. In 2023, the World Health Organization (WHO) released a document on target product profile specifications for readers to be used with rapid diagnostic tests [1]. They defined a reader as “a dedicated hardware instrument or an app that operates on a general-purpose mobile device such as a tablet or phone”, which may be used in screening and diagnostics to support test performance [1]. There is a paucity of research regarding whether the accuracy of HIVST can be optimized by digital supports or solutions. In the post-COVID era, the price of self-tests is on a decline, and the use of digital applications and platforms is on the rise, which shows promise for increasing digital HIVST. Accuracy is estimated using standard metrics of sensitivity (Sn), specificity (Sp), positive predictive value (PPV), and negative predictive value (NPV). In 2012, when the OraQuick In-Home HIV Test was approved by the Food and Drug Administration (FDA).the company reported an average sensitivity rate of 91.7% and a specificity rate of 99.9% [8]. Although studies have reported accuracies ranging from sensitivity of 87.5%-99.5% and specificity of 98%-100% for the OraQuick self-test compared to reference standard in supervised settings [9–11]. These high sensitivity and specificity rates are promising evidence of the reliability of the OraQuick self-test. Despite this, performance of self-tests integrated with digital solutions and their consequent impact on test accuracy has not yet been investigated.

To enhance the HIVST process, an award-winning application (HIVSmart!) was developed by our lab and integrated with the HIV self-testing process to guide patients through their HIVST experience. It also connects patients to clinical care, including counselling, staging, confirmation of test results and treatment initiation. It was extensively tested in South Africa for feasibility, usability, and recently for impact with good results [12]. Between 2017-2019, an original field trial was conducted in South African township populations to explore the impact of the digital strategy on new infections, referrals to test and linkages [12]. Within the context of this trial, we examined using secondary data, the accuracy estimations of oral self-test aided by the HIVSmart! app. HIVSmart! is a prime example of an innovative tool that can be used to improve HIVST readout by unsupervised users. Such solutions should be considered to aid in achieving the UNAIDS 95-95-95 targets by 2025.

Currently, about 20% of all PLWH reside in South Africa, where 7.5 million people are HIV-positive [13]. In 2023, HIVST kits became accessible in South Africa [14]. Due to their popularity, the demand for apps has also risen. Since COVID-19, a greater use of readers and digital applications for rapid point of care and self-tests is being recommended and reviewed by regulatory bodies. The WHO and the Foundation for Innovative New Diagnostics (FIND) released guidelines for product specifications of these supports. In this context, we aimed to examine the diagnostic accuracy estimations with our app-based strategy. This estimation removes the subjectivity experienced by users during self-test interpretation, either in a supervised form with healthcare professionals or unsupervised in a private space. The findings will impact policy and future research for digital self-testing integrated solutions.

### Study objective and hypothesis

The objective of this study was to evaluate the diagnostic accuracy of HIVSmart! for oral self-test result interpretation against the reference standard of two blood-based rapid tests and a lab-based HIV RNA test. We also analyzed the difference in accuracy readouts between the supervised and unsupervised groups of those who self-tested.

We hypothesized that the self-testing method along with HIVSmart! that guides participants through the process of self-testing, result interpretation, together with counselling, will result in a slight improvement in accuracy estimations, documented by the metrics of sensitivity, specificity, PPV, and NPV, currently indicated in the FDA document [8]. As well, we predict that those in the supervised group will receive higher accuracy metrics, considering they are supported by healthcare professionals and the HIVSmart! app, compared to the unsupervised group, that is being performed in a private space.

The primary outcome of this analysis was to explore the accuracy of the HIVSmart! guided self-testing process, supported by the participant-interpreted picture of the self-test result, against the lab-confirmed reference standard test result (collected at baseline, prior to administering the self-test). The secondary outcome was to measure the difference in accuracies reported between the supervised and unsupervised arms of the trial.

## Methods

### Study design

We conducted a secondary data analysis of 1513 participants, with both self-test and reference standard results, enrolled in the intervention self-testing arm of the quasi-randomized controlled trial [12]. The original trial successfully evaluated the clinical and public health impact and effectiveness of the HIVSmart! self-testing strategy on new infection detection (1.5 times), referrals to self-test (5.5 times) and operationalizing linkage to care (99.7%) in township populations in South Africa [12].

### Participants and setting

Participants over 18+ years of age, having an unknown HIV status at baseline, and with access to an Android/iPhone smartphone or the ability to use a tablet/smartphone for self-testing via HIVSmart! were included. Participants meeting the eligibility criteria and presenting for HIV testing at community outreach clinics in Cape Town, South Africa were included. Potential participants were excluded from the study if they were on ART, had a confirmed HIV diagnosis, or had a serious medical condition that required hospitalization. Clinic staff also recruited participants during routine and drop-in visits. Recruited participants were encouraged to refer their partners, friends, and family if they fit the eligibility criteria. As well, community outreach was accomplished by healthcare workers, word of mouth, handouts/flyers, demonstration videos in the clinics, a Facebook page, and radio/television announcements.

All districts in Western Cape Town were geo-mapped and a random number sequence was generated in STATA V.12. Within each of the three geographic sampling frames, two geographically separated clinics were then randomly sampled, and six clinics were selected for intervention and control (standard of care) clinics. Participants were offered the choice of strategy: either supervised performed (under observation by health care professionals) at the clinic, or unsupervised at any private space or location of their choosing, including home, offices, or kiosks.

### Test methods

After informed consent, the participants were allowed to choose between the supervised or unsupervised self-testing strategy. In both strategies, the participants first received a brief introduction to the app-facilitated self-testing process, then they conducted the self-test in the clinic (supervised testing) or other spaces (unsupervised testing). Before the participant left the clinic, blood for reference standard tests was collected and tests were performed at baseline. Two rapid finger-prick blood tests, and an HIV RNA based laboratory test. Pre-test counselling was offered through the HIVSmart! app and participants performed the OraQuick rapid HIV-1/2 self-test (OraSure Technologies Inc, USA) usually within the same day as reference tests. If the participant chose the unsupervised option and took the test home, they were instructed to complete the test and provide results within 24 to 48 hours, which each participant successfully executed. All self-tests were completed with the assistance of HIVSmart!. The self-test interpretation was also performed with the help of HIVSmart! and recorded on the app. To conduct the self-test, the participant collected oral fluid samples by pressing the flat pad of the test kit into their mouth and swabbing around the upper and lower gums, then placing the flat pad into the tube of buffer liquid, and letting it sit for 20 minutes before reading the result.

The HIVSmart! app supported participants through their testing process. Participants were first introduced to the “virtual clinical assistant” who walked them through preliminary information, the HIVST steps, and post-testing support. Before testing, participants were asked to answer a few questions about their sociodemographic characteristics. Once completed, the app provided evidence-based information on what HIV and acquired immunodeficiency syndrome (AIDS) are, how it could be contracted, who may become infected, testing options, why to get tested, what a self-test is, and what to do once your self-test result is received. After this informative pre-test counselling section, the virtual assistant completed a risk assessment evaluation through a small set of questions and provided a result at the end to let the participant know their risk score of acquiring HIV. Details regarding the risk scores generated from study data, are available in recent publications [15,16]. After the risk score assessment, the test procedure was explained by the app-based assistant, and an instructional video of 20 minutes was provided for the participant to conduct the self-test, during which the participants were presented with questions to keep them engaged. These questions referred to their self-testing experience and preferred method of linkage to care and follow-up care.

When the 20 minutes of testing had passed, the app displayed images of the self-test results and asked the participants to choose which most resembled their test result, which mitigated the uncertainty of test interpretation. The user also had the option of scanning the test with the device’s camera and uploading a picture of their self-test result. The test results, when recorded by the tester, were made available to the study coordinator and PIs on the app platform. The self-test results were then also interpreted by the healthcare professional, who was tasked with linkages to care. Self-test results were then compared to the reference tests and HIV status was confirmed for the participant by the counsellor, immediately followed by linkage to post-test counselling options. Linkages included a direct app-built phone line to a counsellor nearby, linked to the University of Cape Town, and options for participant preferred clinics providing care in their region.

A positive self-test result would show two lines on the test device (one control line and one test line), possibly including a faint test line. A negative self-test result would appear as one control line. Invalid test results were defined as the appearance of no lines, and were obtained by few participants, but were not included in the final analysis. If any indeterminant results were found from one of the two rapid tests, a third rapid test was performed before the lab confirmatory test. As well, indeterminate reference standard tests were handled by the phlebotomist and were then repeated, with the repeated test result used in analysis. Of note, the readers of the reference standard test, such as healthcare staff and researchers, were un-blinded to the results of the index test with the app. Clinical information of testers was available to the assessors of the reference standard test only for participants in the supervised group, but not within the unsupervised group. All collected personal data was deidentified and encrypted to conserve the anonymity of participants’ identities throughout the statistical analysis.

### Statistical analysis

Given that the unaided self-test accuracy was reported to be sensitivity 91.7% and specificity of 99%, following FDA reporting, a sample size of 1250 was deemed sufficient for the self-test with HIVSmart! estimations. The secondary data analysis was performed in STATA V.17. We determined the Sn, Sp, PPV, and NPV of the digital HIVST result versus the lab reference standard result. As well, the separate accuracy metrics between the supervised and unsupervised arms were calculated to analyze the impact of supervision on the participant’s test accuracy. These values were then compared to confirm the relationship between self-tests and the reference standard.

### Ethics approval

The study and analyses were approved by the Institutional Review Board of the Research Institute of McGill University Health Centre and the IRB of the University of Cape Town. All participants of the trial gave written informed consent to participate in the study.

## Results

### Participant demographics

The mean age of the participants was 28 years (range: 19-37), a majority of them were female (64.76%). Participants in the supervised arm conducted the self-test at the same time as the reference standard sample was collected at the clinic, while those in the unsupervised arm reported their self-test results within 24 to 48 hours of reference testing. Other demographic and baseline characteristics are presented in the published article of the initial trial data [12].

Of 1535 consenting participants, a vast majority (n=962) chose the unsupervised option for self-testing. However, 22 participants (8 in supervised arm and 14 in the unsupervised arm) were excluded from the analysis, for either their self-test or lab confirmatory result were not available. Further details of the participants can be found in the published article of the initial trial data [12].

### Test results

Comparing the self-test result aided with the digital HIVSmart! app to the lab reference standard, the Sensitivity was 95.52% (95% CI: 94.48%-96.56%), Specificity was 99.93% (95% CI: 99.79%-100.06%), PPV was 99.22% (95% CI: 98.78%-99.67%) and NPV was 99.57% (95% CI: 99.24%-99.90%) (Table 1). No adverse effects were reported with the test strategy. For participants in the supervised strategy, the estimations were Sn: 93.65% (95% CI: 91.64%-95.66%), Sp: 100.00% (95% CI: 100.00%-100.00%), PPV: 100.00% (95% CI: 100.00%-100.00%), and NPV: 99.21% (95% CI: 98.48%-99.94%) (Table 2). Regarding participants in the unsupervised strategy, the estimations were Sn: 97.18% (95% CI: 96.13%-98.24%), Sp: 99.89% (95% CI: 99.67%-100.00%), PPV: 98.57% (95% CI: 97.82%-99.33%), and NPV: 99.77% (95% CI: 99.47-100.00) (Table 3). We compared a digital aided self-test results to the FDA reported sensitivities and specificities. We did not find any statistical significance for overall pooled sensitivity of both arms at 5% significance level with $\chi^{2}$-values of 0.9495 and 0.3831, and p-values of 0.4203 and 0.5259, respectively. However, we found the reported sensitivity in the unsupervised arm to be 97.18% and specificity at 99.89%. Which demonstrates that the sensitivity is massively improved by 5.4%, which translates to a reduction in interpretation of false negative tests.

**Table 1:** ST+HIVSmart! versus lab-confirmed HIV for participants.

		Lab-Confirmed HIV		Total
		+	–	
ST+HIVSmart! Result	+	128	1	129
	–	6	1378	1384
Total		134	1379	1513
Sn (%)		95.52, 94.48-96.56 (95% CI)		
Sp (%)		99.93, 99.79-100.00 (95% CI)		
PPV (%)		99.22, 98.78-99.67 (95% CI)		
NPV (%)		99.57, 99.24-99.90 (95% CI)		

**Table 2:** Supervised arm performance: ST+HIVSmart! vs. lab-confirmed HIV status.

		Lab-Confirmed HIV		Total
		+	–	
ST+HIVSmart! Result	+	59	0	59
	–	4	502	506
Total		63	502	565
Sn (%)		93.65, 91.64-95.66 (95% CI)		
Sp (%)		100.00, 100.00-100.00 (95% CI)		
PPV (%)		100.00, 100.00-100.00 (95% CI)		
NPV (%)		99.21, 98.48-99.94 (95% CI)		

**Table 3:** Unsupervised arm performance: ST+HIVSmart! vs. lab-confirmed HIV status.

		Lab-Confirmed HIV		Total
		+		–
ST+HIVSmart! Result	+	69	1	70
	–	2	876	878
Total		71	877	948
Sn (%)		97.18, 96.13-98.24 (95% CI)		
Sp (%)		99.89, 99.67-100.00 (95% CI)		
PPV (%)		98.57, 97.82-99.33 (95% CI)		
NPV (95%)		99.77, 99.47-100.00 (95% CI)		

Tabulated results are illustrated below:

## Discussion

### Key findings

Accuracy of self-tests is reported by sensitivity and specificity parameters. Pooled sensitivity of both arms of the strategy (oral-based OraQuick self-test aided by HIVSmart! app-based readout) was 95.52% (95% CI: 94.48-96.56), and specificity was 99.93% (95% CI: 99.79%-100.00%). Break up wise: the unsupervised self-testing strategy reported a slightly higher sensitivity of 97.18% (95% CI: 96.13-98.24) vs. the supervised strategy at 93.65% (95% CI: 91.64%-95.66%). Specificity of the supervised strategy was perfect at 100% (95% CI:100.00%-100.00%) vs. unsupervised at 99.89% (95% CI: 99.67%-100.00%). In 2012, when the FDA approved OraQuick, reported sensitivity was 91.7% and specificity was 99.9% [8]. These were reported with a naked eye readout of the oral test without any digital support. In our field trial, we found an increase in both parameters with the use of digital support app as in a pooled sensitivity of both arms at 95.5% and specificity of 99.9%-100%. In both cases, the unsupervised and pooled sensitivity were higher than un-aided self-testing reported by the FDA, making a case for a greater use of digital supports to improve the confidence in readout of self-test results. To sum up, sensitivity is a key parameter for it allows us to be safe in knowing that the positive test results are accurate; and on that front, digital innovations can help optimize a positive test readout, and sensitivity estimations, thereby preventing test misclassification. With the use of the app, both the positive and negative predictive values increased significantly to 99.22% (95% CI: 98.78%-99.67%) and 99.57% (95% CI: 99.24%-99.90%) respectively. These values (PPV and NPV) depended heavily on prevalence. In South Africa, prevalence of HIV is high (>5%), so this finding can convince testers that in such a setting, a positive test by both app and oral test, is truly positive and the use of the app-based readout together with the self-test improves the reliability of self-testing. Conversely, in a low prevalence setting, the likelihood of the combined app-based readout and negative oral test, is likely to indicate a negative test result.

Dividing the results to analyze the supervised and unsupervised arms individually yielded very fascinating results. The specificity, PPV, and NPV were all moderately similar, but the sensitivity was 93.65% for the supervised participants, compared to 97.18% for unsupervised participants. This is an interesting result, where unsupervised arm reports an increase in 3.53% sensitivity considering it is likely to be assumed that one would conduct a self-test more precisely with the supervision of a healthcare professional. A possible explanation for this outcome is that the participants who chose the unsupervised option were more comfortable in their ability to conduct the self-test alone with the digital app, and the presence of a healthcare professional did not help that much. As supported by past research, participants in healthcare research can dislike practitioners, especially in the case of HIV due to stigma and judgement [17]. These results mean it is possible to infer that the use of HIVSmart! on its own, without any practitioner support, is sufficient in supporting patients through the self-testing process and negates any anxiety some may feel with healthcare workers.

Given the novelty of self-testing digital innovations, it is not surprising that previous studies have shown intriguing results regarding HIVST accuracy, but not analyzed the impact that digital supports have on test accuracy. For example, one cross-sectional study conducted by Martinez Pérez et al. investigated the accuracy of unaided, no digital support based, OraQuick In-Home HIVST in rural populations in South Africa [18]. Participants completed the oral self-test under the supervision of a counsellor, followed by blood based rapid tests (Determine^TM^ and Unigold^TM^) serving as the reference standard. Reported sensitivity was 98.7% (95% CI: 96.8%-99.6%) and specificity was 100% (95% CI: 99.8%-100%). PPV and NPV were determined to be 100.0% (95% CI: 98.2%-99.9%) and 99.7% (95% CI: 99.4%-99.9%) respectively [18]. These high values are supportive evidence of the OraQuick test’s field accuracy; however, these tests were conducted under the supervision of a counsellor and were compared to rapid blood-based tests instead of a lab-based result. It can be safely assumed that the accuracy metrics could potentially decrease if the self-tests were conducted alone, but with the support of digital interventions, these metrics can be maintained or even increased. In real-life settings, self-tests are performed privately and especially when it comes to the stigma of HIV, the autonomy of conducting a self-test in one’s preferred privacy setting is an important aspect. To protect this sense of autonomy and the high self-test accuracy in professional settings, digital innovations can safely increase the confidence in the readout of self-test result.

In another study, Peck et al. found that less than 25% of participants were able to conduct the self-test correctly and about 47.3% of all participants made multiple errors, when unsupervised [19]. These were also performed without digital supports like mobile apps or websites. In terms of self-test result interpretation, only 79.7% of negative results and 78.7% of strong positive results were correctly interpreted [19]. Unfortunately, only 26.7% of the faint positive results were correctly interpreted by the participants who received this result ([19]. In a third study by Ng et al. that analyzed the accuracy in the OraQuick self-tests performed by untrained individuals alone compared to with trained healthcare workers [20]. The untrained individuals performed the test with an 11-step package that was designed by the study team and replaced the OraQuick test insert. The package included instructions on kit preparation, collection of oral fluids, specimen insert into the buffer fluid, and result interpretation. As well, a sheet with seven images of the possible ranging results were provided to participants, likely increasing result interpretation. This study found the self-test sensitivity and specificity to be 97.4% and 99.9% respectively [20]. Importantly, the k-value for inter-rater agreement between the self-test and test with healthcare worker was found to be 0.97, meaning their test results were similarly interpreted by the participants conducting the self-test alone and healthcare workers. These results are very similar to what we obtained in our study, implying that the pictorial interpretation allows for improvement in accuracy readout compared to unsupported test result interpretation.

Finally, Stevens and colleagues reviewed global data on accuracy (sensitivity and specificity) of OraQuick Rapid HIV-1/2 self-test and other self-tests, prior to 2015 and reported a median pooled sensitivity of 93.6% and specificity of 99.9% respectively [21]. However, it is notable that one of the studies from this review did not include faint or weak positive lines as positive test results. Based on these findings, it appears the specificity of these tests has remained stable over time, but the sensitivity merits an improvement that can only go so far because oral mucosal transudate cannot be as sensitive as whole blood. This systematic review also noted the varied abilities of participants in performing oral- and blood-based self-tests [21].

### Implications for research

WHO and FIND recommend using readers, apps, and digital supports to help optimize the quantitative/qualitative readout of rapid tests in field settings [22]. In this context, our findings support potential for a promising method of readout via HIVSmart!.

### Implications for practice

The use of digital innovations together with self-tests improve the accuracy and predictive value. This can increase confidence in the self-test results and aid acceptance and implementation. HIVST and the HIVSmart! app and related digital supports, should be viewed as complementary means to current clinical processes and practices. Once a self-test result is obtained, the patient may choose to seek confirmatory tests, through the app, if they desire. Considering the high specificity rate of this test together with the app-based readout, those with negative results will likely not require a confirmatory result, therefore saving laboratory testing supplies for those who are suspected to be HIV-positive. As well, the accessibility and sense of autonomy that are associated with self-testing and HIVSmart! will likely encourage patients to recommend this testing option to others in their social circles, hopefully increasing the uptake of HIVST and in turn, increase serostatus awareness. With user consent, the use of HIVSmart! can allow self-test results to be safely recorded for data collection, which may benefit future educative applications to further increase the accuracy of self-test interpretation by patients.

### Strengths

The originality of this work will pave the way for more research in support of digital innovations and readers to support the use of self-tests for infectious diseases in the near future. HIVSmart! is a promising digital innovation that allows patients to choose preferred language options, watch simple videos for testing directions, and access continuative care directly through the application as soon as their final test results are relayed. In addition, data collection via HIVSmart! is beneficial for further studies to guide in improving self-tests and interpretation by patients. In the future, we may be able to expand on this concept to use molecular tests and readers that would remove the subjectivity experience in interpretation entirely.

### Limitations

In real life, it is not possible to repeat self-tests given that it would inflate the cost of self-testing. An example of this is that the participants only conducted the self-test once, which may hinder the reliability of the test results. On the other hand, repeating the self-tests could have improved users’ testing method and using only the second-test results could have produced a lower number of indeterminate tests, which could increase the accuracy parameters. It is difficult to say what would happen if the self-tests were repeated, but for test-retest reliability, this should have been considered if viable.

Finally, another possible limitation is related to the generalizability of these results. The study population consisted of populations from townships, who are at risk of acquiring HIV, therefore these results may not be applicable to larger general populations.

Despite the study limitations, a major benefit of the study was that the flexibility and choice of venue maintained with a high participant engagement and resulting in a linkage to care of 99% and retention of 97% which is the highest to date reported with app-based programs. The high rate along with ease of accessibility and flexibility, and high accuracy increases the likelihood of acceptance of this solution.

## Conclusion

Our digital HIVST strategy reported an improvement in the pooled sensitivity of 95.5% (95% CI: 94.48%-96.56%) (from 91.7% FDA approval of self-test), together with a high specificity of 99%-100%. High positive and negative predictive values of nearly 99%-100% demonstrate that app-based digital interpretation removed subjectivity, increased accuracy of test result interpretation, and allowed for test result recording, as well as storage of data for monitoring purposes. The difference in accuracy parameters between supervised vs. unsupervised arms supports the possibility of users partaking digital HIVST vs. unaided HIVST. The stress and stigma of observation sometimes impairs performance in clinics. Mobile apps and readers can be useful adjuncts to improve the accuracy estimations of self-tests, obliviating the need for further testing and catalyzing the clinical rapid action plan of ART initiation. These data are relevant for machine learning based interpretations of HIVST and encourage the future use of digital HIVST strategies, worldwide wherever feasible.
